# Emerging Data on the Diversity of Molecular Mechanisms Involving C/D snoRNAs

**DOI:** 10.3390/ncrna7020030

**Published:** 2021-05-06

**Authors:** Laeya Baldini, Bruno Charpentier, Stéphane Labialle

**Affiliations:** Campus Brabois Santé, Université de Lorraine, CNRS, IMoPA, F-54000 Nancy, France; laeya.baldini@univ-lorraine.fr

**Keywords:** box C/D small nucleolar RNA, C/D snoRNA, small nucleolar ribonucleoprotein, snoRNP, C/D snoRNP biogenesis, nucleolus, molecular biology

## Abstract

Box C/D small nucleolar RNAs (C/D snoRNAs) represent an ancient family of small non-coding RNAs that are classically viewed as housekeeping guides for the 2′-O-methylation of ribosomal RNA in Archaea and Eukaryotes. However, an extensive set of studies now argues that they are involved in mechanisms that go well beyond this function. Here, we present these pieces of evidence in light of the current comprehension of the molecular mechanisms that control C/D snoRNA expression and function. From this inventory emerges that an accurate description of these activities at a molecular level is required to let the snoRNA field enter in a second age of maturity.

## 1. Introduction

On several occasions, the fortuitous identification of RNAs with odd features turned out to be at the origin of a new family of non-coding RNAs (ncRNAs). A perfect example is the characterization of development timing mutants in *C. elegans*, leading to the identification of tiny ncRNAs originally named small temporary RNAs (stRNAs) lin-4 and let-7 [1,2]. These two RNAs were, in fact, part of the microRNAs class, which now has more than 1000 members. The discovery of the box C/D small nucleolar RNAs (C/D snoRNAs) is another striking case. Abundant low molecular weight RNAs were first identified in nuclear [3] and nucleolar fractions [4,5]. Nucleolar U3 RNA was later demonstrated contributing at an early stage of the ribosomal RNA (rRNA) processing pathway by acting as a chaperone folding the nascent primary transcript for the first endonucleolytic cleavages [6,7,8,9]. Next, U3 conserved sequences and common associated proteins were subsequently identified for other nucleolar ncRNAs leading to classify hundreds of snoRNAs with U3 in the family of the box C/D snoRNAs [10,11,12,13,14]. A large number of the box C/D snoRNAs were shown forming small nucleolar ribonucleoproteins (snoRNPs) to participate in ribosome biogenesis (for recent reviews, see [15,16,17,18,19]). However, U3 snoRNP appeared singular since most of the box C/D snoRNAs are components of RNP enzymes catalyzing the site-specific 2′-*O*-methylation of functionally important regions in rRNA. Concomitantly, a second large family of snoRNAs with conserved sequences boxes H and ACA were discovered [20,21,22] (for reviews, see [15,23]). The box H/ACA snoRNAs form snoRNPs that are RNA-guided modification enzymes targeting uridines for their isomerization into pseudouridines. As for box C/D snoRNPs, a few H/ACA snoRNAs are also involved in pre-rRNA processing machinery [24,25] (for reviews, see [15,16,18,19]). Subsequently, small Cajal Bodies RNAs (scaRNAs) with similar features as snoRNAs and forming equivalent catalytic RNPs were found enriched in Cajal bodies (CBs, for a review, see [26]). In these nuclear bodies, some are the catalysts for ribose 2′-*O*-methylation and for pseudouridylation inside functionally important sequences of spliceosomal small nuclear RNAs (snRNAs) (for a review, see [27]). Hence, RNA-guided modification is not the prerogative of snoRNPs, and scaRNPs illustrated a potential of diversity towards substrate specialization depending on their nuclear localization.

Data accumulated this last decade added diversity to the pattern of expression, the place, the partners, and the functions of snoRNAs that could not still be perceived as confined to ribosome-associated functions. On the other hand, several of these RNAs still have no identified function, while some of them have been identified as key actors in human pathologies. Here, we compare novel features recently discovered for box C/D snoRNPs with the conventional ones that are firmly established. We focus on the box C/D sno/scaRNAs and their associated RNPs as more structure, and function diversity is up to now identified among this subclass compared with box H/ACA sno/scaRNPs. Importantly, complementary and detailed information on the (patho)physiology of both C/D and H/ACA snoRNAs, including in vivo knockout models, are available in recent reviews [15,28,29,30,31,32,33].

## 2. Molecular Features for the Canonical Paradigm of RNA-Guided Activity: The Box C/D RNP Methyltransferases

It is probable that eukaryotic snoRNAs originate from an ancestor present before the separation between Eukarya and Archaea (for a review, see [34]). Indeed, small RNAs with box C/D or box H/ACA snoRNA features, named sRNAs, are also acting for RNA-guided modification in archaeons [35,36,37,38,39,40,41] (for a review, see [42,43]). This phylogenetic feature highlights the essentiality of the cellular activity of these systems that could have expanded in eukaryotic cells with other functions and mechanisms of action.

**Figure 1:**
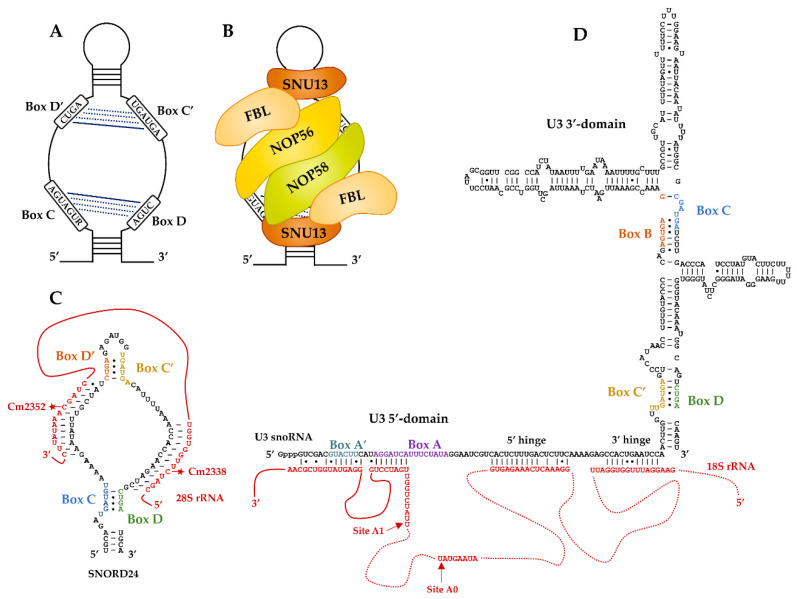
Schematic overview of guide box C/D small nucleolar ribonucleoproteins (C/D snoRNPs) and their target RNAs. (**A**) Box C/D small nucleolar RNAs (C/D snoRNAs) contain conserved sequences called C/D and C′/D′ boxes that fold into a kink-turn (K-turn) containing Watson–Crick pairings (solid blue lines) and non-canonical pairings U:U, G:A, and A:G (dotted blue lines). (**B**) Conventional C/D box snoRNPs associate a C/D snoRNA with the core proteins Nop1p/Fibrillarin (FBL), Nop58p/NOP58, Nop56p/NOP56 and Snu13p/SNU13 in *Saccharomyces cerevisiae* and Human, respectively. FBL is the methyltranferase that catalyzes the 2′-O-methylation of target RNAs. (**C**) Schematic representation of the bidimensional human SNORD24 structure (adapted from [50]). This snoRNA guides the 2′-O-methylation of 28S rRNA at positions C2338 and C2352. Classically, the methylated nucleotide (red star) hybridizes with the nucleotide located at the fifth position upstream of the D and D′ box sequences. (**D**) Schematic representation of the bidimensional U3 snoRNA structure and its association with pre-18S rRNA in *Saccharomyces cerevisiae* (adapted from [61,62]). U3 contains two C/D-like motifs called C′/D and B/C boxes that fold into a K-turn recognized by the Snu13p core protein [14]. In addition, the B/C motif recruits Rrp9p (U3-55K in humans) that is essential for 18S rRNA processing. The A/A′ motifs and the 5′/3′ hinges, located in the 5′ terminal domain of U3, hybridize with the 18S rRNA (solid red lines) and the 5′ ETS (external transcribed spacer, dotted red lines) of the 35S pre-rRNA, respectively. The base-pairing is located near the A0 and A1 pre-rRNA cleavage sites and is essential for the processing of 18S rRNA.

The box C/D snoRNAs have an average length of 60–90 nucleotides and are folded as a single irregular hairpin structure. They possess conserved sequence patterns forming a box C (consensus sequence 5′-RUGAUGA-3′ where R = purine) and a box D (consensus sequence 5′-CUGA-3′), which are located at the 5′- and 3′-ends of snoRNAs, respectively. In most cases, additional boxes designated C′ and D′ are present in the internal region of the transcript; however, these sequences are less well conserved and sometimes degenerated [11,44]. In the secondary structure of the snoRNA transcripts, the conserved boxes are brought together and form the C/D and C′/D′ motifs (Figure 1A). The C/D motif corresponds to a structural element organized as a kink-turn (K-turn), which is characterized by a 3-nucleotide asymmetric internal bulge surrounded by two stems I and II and comprising at the edge of stem II tandem G:A and A:G sheared base pairs. In the 3D structure, a sharp bend of the ribose-phosphate backbone is formed that is stabilized by stacking interactions and featuring a protruded uridine invariably present in the internal budge [14,45,46,47] (for a review, see [48]). This type of motif is also present in U4 snRNA [14,49,50] and in both box C/D and box H/ACA archaeal sRNAs [38,51,52]. The motif C′/D′ in archaeal sRNAs is organized as a K-loop lacking stem I and closed by a terminal loop [53]. The box C/D snoRNAs K-turn motif constitutes a specific binding site for Snu13p/SNU13 (in Yeast and Human, respectively) that nucleates RNP formation through the subsequent recruitment of Nop58p/NOP58 and the SAM-dependent 2′-O-methylase Nop1p/FBL [14,54,55]. The C′/D′ motif constitutes a secondary Snu13p/SNU13 binding site leading to the assembly of a second half-particle through recruitment of the Nop56p/NOP56–Nop1p/FBL module [50,56,57] (Figure 1B). Based on structural studies performed on the archaeal components [58,59] (for a review, see [15]), it has been proposed that the sub-RNPs assembled on each RNA motif are connected through the coiled-coil dimer of paralogous Nop56p/NOP56 and Nop58p/NOP58. Recent reconstitution of C/D snoRNP particle from the thermophilic yeast *Chaetomium thermophilum* indicates that an in vivo assembly machinery allows the specific assembly of Nop58 and Nop56 on the C/D and C′/D′ motifs, respectively [60].

Guide activity of box C/D snoRNAs relies on the sequence located immediately upstream of boxes D and D′ that forms base pairs with the RNA substrate and specifies the site of ribose 2′-*O*-methylation. The modified residue is the fifth nucleotide upstream of the D or D′ sequences [11,63] (Figure 1C). Information on the molecular bases for the positioning of the active site of the catalytic subunit of the RNP was gained by structural studies performed with reconstituted archaeal box C/D RNPs [58,64]. The sequence potentially available for substrate binding usually has a length of 10 to 21 nucleotides [11,65,66]. One study based on 3D structures of artificial yeast sRNPs hybridized to RNA substrates proposed that a maximum of 10 bp can be accommodated by such particles [67]. Conversely, a subset of *Saccharomyces cerevisiae*, human, and *Arabidopsis thaliana* box C/D snoRNAs were shown to have extra base-paired regions leading to enhanced methylation [44,68,69].

## 3. U3, the First Identified Atypical Box C/D RNP

As mentioned in the introduction, U3 has atypical features. First, although it contains the 2′-*O*-methyltransferase Nop1p/FBL, the U3 snoRNP has never been shown to be a catalyst for RNA 2′-*O*-methylation. Second, the U3 snoRNA contains four phylogenetically conserved sequences that form two non-conventional box C/D-like motifs named C′/D and B/C (Figure 1D). Each forms a K-turn structure recognized by Snu13p/SNU13 [14,70,71,72]. However, protein assembly on these two motifs is dissymmetric. A specific U3 core protein Rrp9p in yeast and U3-55K in human [73,74,75] is recruited on the B/C motif in a Snu13p/SNU13-dependent manner [72,76,77,78] together with Nop56p/NOP56 and Nop1p/FBL [79,80]. In contrast, the classical set of C/D box proteins Snu13p/SNU13, Nop58p/NOP58, and Nop1p/FBL is recruited on the C′/D motif [14,81]. Recent cryo-EM structures from *S. cerevisiae* and *Chaetomium thermophilum* confirmed this pseudo-symmetric organization of U3 snoRNP [61,82,83,84].

Compared with other C/D snoRNAs, U3 has a long 5′-terminal region needed for the function of the particle during pre-rRNA processing [7,85,86,87,88,89]. Pre-rRNA maturation begins in terminal knobs corresponding to the packaging of the nascent rRNA primary transcript into a large structure, named small subunit (SSU) processome. The U3 snoRNP contributes to the assembly of the SSU processome [9]. The U3 snoRNA plays a central role by forming base-pair interactions with several sequences of the 5′ external transcribed spacer (5′-ETS) and the 18S rRNA (Figure 1D). It is viewed as an organizing chaperone to RNA folding into 5 distinct helices that potentiate early cleavage at sites A0 and A1 in the 5′-ETS. The occurrence of helices was investigated by biochemical and genetic approaches [7,62,85,86,87,88,89]. The cryoEM structures confirmed that several of them are formed within the processome [61,82,83,84].

## 4. Biogenesis of Box C/D RNP: Assembly and Nuclear Journey

**Figure 2:**
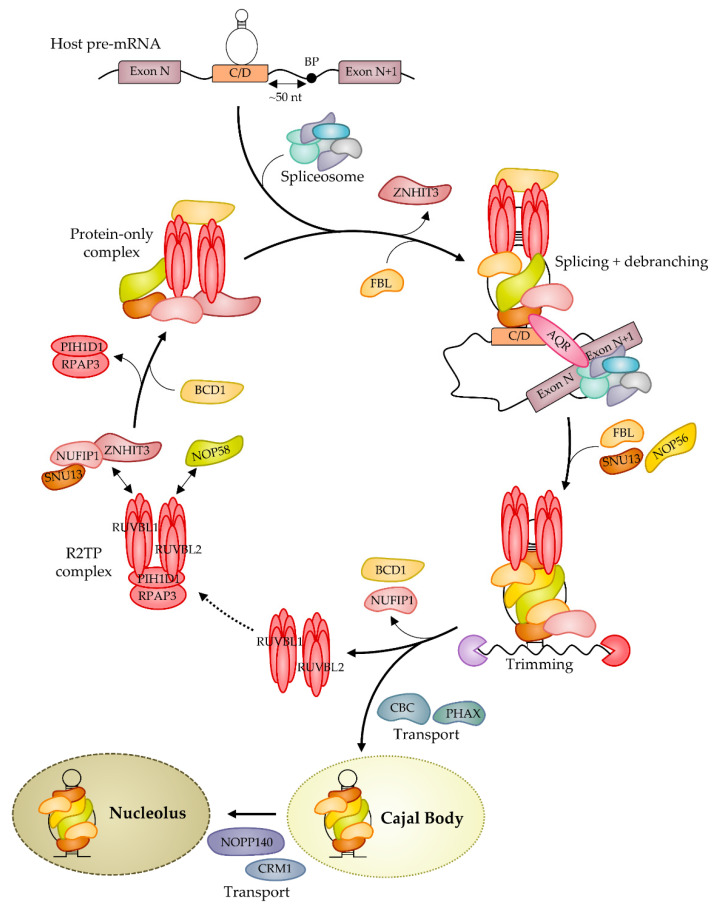
Model of eukaryotic C/D snoRNP biogenesis. The loading of the core proteins SNU13, NOP58, NOP56, and FBL does not occur autonomously in eukaryotic cells but is mediated by several assembly factors. The R2TP complex contains the proteins RUVBL1, RUVBL2, RPAP3, and PIH1D1 and the latter interacts directly with NOP58 to stabilize it. The assembly platform NUFIP1 and its stabilizing factor ZNHIT3 interact with SNU13 to form a trimer. The essential contribution of the assembly factor BCD1 is less clear; it is integrated in a network of interactions involving PIH1D1, NUFIP1, RUVBL1&2, NOP58, and SNU13, which suggests that these proteins form a protein-only pre-snoRNP complex that scaffolds SNU13 and NOP58 core protein assembly. The proteins are then loaded on nascent snoRNA molecules in a co-transcriptional way upon the direct binding of SNU13 to the K-turn structures formed by the conserved C/D motifs. The efficient RNP assembly of intronic snoRNAs is favored by the association of the splicing and biogenesis machineries via the splicing factor AQR. The nucleation of the pre-snoRNP protects the snoRNA from extensive trimming by exonuclease activities. When released, the assembly factors might be recycled in a new biogenesis process (dotted line). Finally, the C/D snoRNPs are transported to CBs and nucleoli for functional purposes. This model is valid in both *Saccharomyces cerevisiae* and Human.

Biogenesis of snoRNPs is a complex process involving trans-acting factors and starting on nascent transcripts in a co-transcriptional fashion [15,33,90] (Figure 2). In most organisms, snoRNAs are transcribed as long precursors by RNA pol II that are trimmed by ribonucleases at 3′- or both 5′- and 3′-ends to generate mature snoRNAs (for a review, see [30]). Early recognition of snoRNA elements by core RNP proteins protects RNA from degradation during the processing. The specific mode of processing is determined by the genomic organization. In vertebrates, a subset of snoRNAs is encoded by independent transcriptional units, but the majority are embedded in introns of host protein-coding genes. In contrast, to box H/ACA snoRNAs, most box C/D snoRNAs are present at a conserved position upstream of the 3′ splicing acceptor site [91,92], and an optimal distance of ~50 nucleotides upstream of the branch point contributes to efficient box C/D snoRNP assembly [93] (for a review, see [94]). This situation favors splicing-dependent assembly of intronic box C/D snoRNPs and the general splicing factor Aquarius AQR (alias Intron Binding Protein IBP160 [95]) was shown to aid RNP assembly [96]. However, some intronic C/D snoRNA genes are found at divergent locations, such as the human repeated snord116 genes that are each sited ~250 bp after the 5′-splice site of the host gene. Then, considering independent snoRNA-encoded transcripts like the U3 snoRNA, the presence of the core proteins was shown to be linked to 3′-end processing and transcription termination [97,98].

Contrary to archaeal sRNPs [37], the assembly of eukaryotic snoRNPs is not a mechanism that occurs autonomously in vitro. Independently of the coupling with splicing machinery as described above, biogenesis requires trans-acting factors involved in RNP assembly and in nuclear trafficking before the mature RNP reaches the nucleolus. Three independent modules Rsa1p:Hit1p/NUFIP1:ZNHIT3 [98,99], the R2TP complex [100,101,102] and Bcd1p/BCD1(ZNHIT6) [90,101,103,104] were identified contributing to snoRNP assembly. Inactive pre-RNPs are first assembled on nascent transcripts before reaching the CBs where they become mature and functional (for a review, see [33,105]). A model has been proposed postulating that the formation of human box C/D snoRNPs requires the formation of an RNA-free pre-complex composed of the two core proteins SNU13 and NOP58 as well as five assembly factors NUFIP1, ZNHIT3, BCD1, RUVBL1, and RUVBL2 [99]. Rsa1p/NUFIP1 is a platform protein stabilized by the factor Hit1p/ZNHIT3 that forms a tertiary complex with Snu13p/SNU13 [71,99,101]. The two ATPases associated with diverse cellular activities (AAA^+^) RUVBL1 and RUVBL2 (RUVBL1/2, alias TIP49, and TIP48) are members of the complex R2TP, which also includes PIH1D1 (Pih1p or Nop17p in yeast) and RPAP3 (Tah1p in yeast) [100,106,107,108]. This complex cooperates with the chaperone HSP90 and participates in the stabilization and the recruitment of NOP58 on the RNP by direct interaction with PIH1D1 [109]. NUFIP1 interacts with SNU13 during the first stages of maturation and blocks the catalytic activity of the complex [99]. ZNHIT3 is then released, and the C/D box snoRNA, as well as FBL, are recruited before binding of the second molecule of FBL and NOP56. NUFIP1 dissociates from the complex, generating a rotation of the catalytic module of FBL, leading to an active C/D box snoRNP complex. Several proteins manage the nuclear trafficking of the pre-snoRNPs, such as CBC and PHAX for the transfer to CBs (or WDR79 [110,111] and TDP-43 [112] for the scaRNPs) and NOPP140 and CRM1 that control the nucleolar localization [33,113]. All categories of snoRNA traffic through CBs [114]. The cap m7Gppp is a key determinant for U3 to reach CBs, and after hypermethylation, CREM intervenes for reaching nucleoli [115,116] (for reviews, see [113,117]).

## 5. The Diversity of C/D snoRNA Maturation Forms

**Figure 3:**
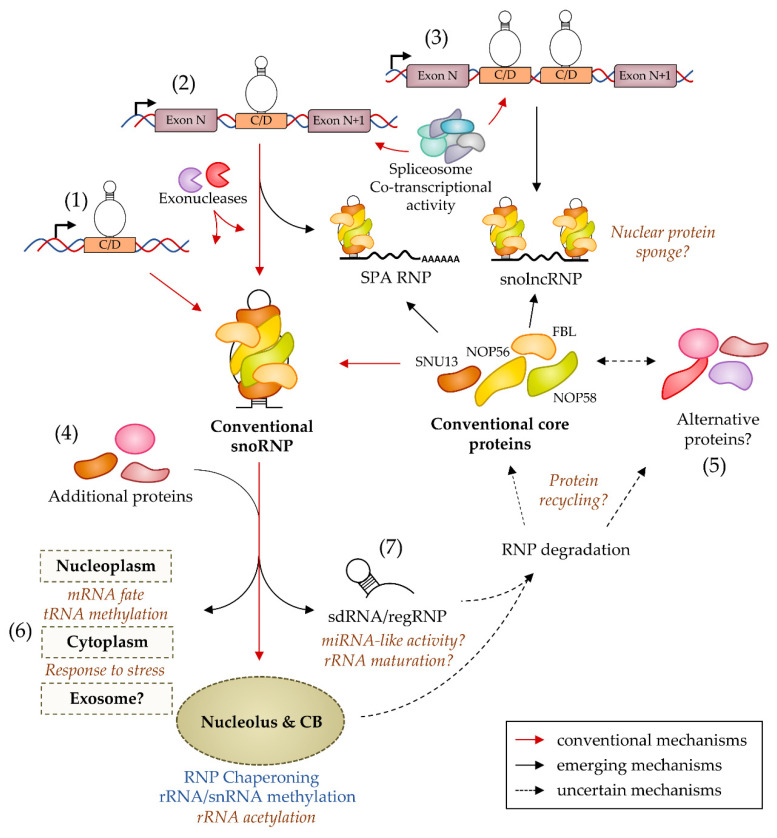
The emerging diversity of C/D box snoRNA processing and of C/D snoRNP biogenesis and function. Precursor C/D box snoRNAs are produced by both independent (**1**) and intronic (**2** and **3**) genes, then processed by the combined action of intron lariat debranching in the case of intronic snoRNAs and exonucleotidic activities acting at both the 5′ and 3′ ends. Co-transcriptional recruitment of snoRNP core proteins on the snoRNA allows the correct processing and biogenesis of the C/D snoRNP. Then, the particle reaches the Cajal body (CB) and the nucleolus to perform activities on snRNAs and rRNAs (in blue). In addition to these conventional mechanisms, C/D snoRNPs perform new activities (in gold italics) linked to the formation of lncRNAs flanked by snoRNA sequences at one or both ends generated from partially-processed, snoRNA-hosting introns (**3**), to the recruitment of additional proteins conferring new properties and/or enzymatic activities (**4**) or to the exchange of conventional core proteins by alternative proteins (**5**), to the trafficking to new subcellular locations (**6**), and to the generation of snoRNA-derived fragments (sdRNAs) or regulatory ribonucleoproteins (regRNPs; **7**).

Precursor snoRNA processing accepts different outcomes, at least in Mammals. First, the pre-snoRNA could be only partially matured to generate long non-coding RNAs (lncRNAs) flanked by snoRNA sequences at one or both extremities (Figure 3). In humans, these RNA species were first described as products of the tandemly repeated snord116 genes at the imprinted PWS locus. This cluster is transcribed as one (or several) precursor RNA(s) whose introns host the snord116 sequences. Intron processing generates conventional snord116 snoRNPs [118] that reach the nucleolus, while the spliced host transcripts accumulate near the transcription site [119]. However, some molecules may undergo unusual processing when certain exons are not spliced, leaving two consecutive snoRNAs that are then trimmed only at the 5′-end for the first and 3′-end for the second. Thus, these so-called snolncRNAs are stabilized by two snoRNP structures at their extremities [120]. Subsequently, snord116 repeats were found to generate lncRNAs containing a 5′-end snoRNP and a polyadenylated 3′-end, called SPA (5′ snoRNA capped and polyadenylated lncRNA [121]). The works characterizing both lncRNAs also proposed that they act as protein factor sponges to control mRNA splicing and gene expression (Figure 3). Since then, several other lncRNAs harboring either C/D or H/ACA snoRNA sequences at their extremities have been described in human cell lines [120]. The nucleolar-specific lncRNA called LoNA is proposed to sequester the core protein FBL via its snoRNA-like 3′-end, which in turn modulates rRNA methylation levels [122]. In addition, the splicing of the human Nop56 pre-mRNA is controlled by an intron-hosted orphan snoRNA called snord86 whose structuration restricts the usage of a nearby splice site. Consequently, an excess of NOP56 protein is expected to favor the production of a cytoplasmic RNA capped at its 5′-end by the snoRNA snord86. This unusual lncRNA is generated at the expense of Nop56 mRNA production, which may constitute a negative feedback mechanism regulating NOP56 protein expression [123]. It is interesting to note that in every case the production of a conventional snoRNA is detected, which opens the possibility that the two maturation processes are in competition or that the snolncRNAs behave as stable intermediate for snoRNA synthesis.

Conventional C/D snoRNAs are also suspected of exhibiting diversity in size. Two distinct isoforms of human C/D snoRNAs differing in their terminal stem length were reported in both normal and cancer cell lines [124]. Such variations involving ± 1–2 bp at the extremities of the basal stem have been recurrently observed by us and others. More surprisingly, the short forms were proposed to form non-canonical snoRNPs based on their lower sensitivity towards NOP58 downregulation. Unfortunately, a precise characterization of these alternative RNPs and the molecular mechanisms that direct their biogenesis has not been reported yet. More surprisingly, the generalization of RNA seq analyses has unveiled another category of snoRNA gene products in the form of short fragments ranging from ~ 20 to 100 nucleotides in size that are collectively called snoRNA-derived RNAs (sdRNAs) or processed snoRNAs (psnoRNAs) (Figure 3). These sdRNAs have been reported in several organisms such as Yeast [125], Protozoans [126], Angiosperms [127], Human [128,129], and Viruses [130] and also include scaRNA derivatives [129,131]. In Human, sdRNAs have been particularly associated with cancers [132,133], and, more largely, they seem to characterize stress conditions such as evidenced in *Saccharomyces cerevisiae* [134] and Wheat [127]. Theoretically, the origin of sdRNAs could derive from regular snoRNAs or being produced directly from precursor RNAs. However, some observations favor the first hypothesis as sdRNAs largely correspond to sequences present in mature snoRNAs and not to surrounding regions. In addition, a study identifying a strong enrichment of sdRNAs corresponding to both extremities of the snord44 gene in HeLa cells reported that their expression was dependent on the core proteins FBL and NOP58 [135]. The relative expression of snoRNAs and associated sdRNAs seems to vary quite independently in *Saccharomyces cerevisiae* [134] and in Human [136], suggesting that they are not simply produced by snoRNA turnover. Nevertheless, the mechanisms of sdRNA biogenesis and regulation are poorly understood. Interestingly enough, FBL has been recently shown to exhibit an RNase activity regulated by the presence of phosphatidic acid [137], which opens the possibility that snoRNA processing is an intrinsic property of C/D snoRNPs upon specific stimuli. The interaction of sdRNAs with proteins is largely unknown, except for a small subset of sdRNAs that associate with proteins of the Argonaute family and harbor microRNA-like [129,138] or piRNA-like [139] properties. The biogenesis of the formers, also called sno-miRs, has been partially evaluated. Based on few studies, the generation of the microRNA-like fragments (also called sno-miRs) requires [129,138] or not [140] the RNase III activity of DICER and are dependent [141,142] or not [140] on the RNase III activity of DROSHA. It could be expected that the study of additional sno-miRs—if they exist [143]—could clarify the mode(s) of biogenesis. Conversely, it is not known whether most of the sdRNAs reported under various cellular conditions are functional or part of the degradome, which leads to a little-addressed question: How and when are snoRNAs degraded, and how is this regulated? To date, we have no answers, although they are crucial for understanding the fate of snoRNPs.

## 6. The Multiple Regulations of C/D snoRNP Biogenesis

In Mammals, the expression of intronic snoRNA genes could be uncoupled from the expression of host genes, e.g., due to alternative splicing and nonsense-mediated decay [144]. Originally, the fact that, in general, host genes exhibit a high transcription rate has suggested that an important regulation step of snoRNA expression occurs at the step of RNP biogenesis. The sophistication of eukaryotic C/D snoRNP assembly involving dedicated factors may represent a site of opportunity for regulation. In agreement, N6-methylation of adenine could interfere with K-turn formation and Snu13p/SNU13 binding, thus preventing the first step of protein assembly on the nascent snoRNA in both Yeast and Human [145,146]. Moreover, several post-translational modifications of core proteins have been identified: NOP58 stability has been shown to depends on sumoylation [147,148], and the stabilization of FBL, NOP58, and NOP56 by O-GlcNAcylation is essential for snoRNP assembly [149]. However, the elucidation of the pathways controlling these modifications is pending. The formation of the chaperone complex R2TP is dependent on nutriment availability that controls, via the mTOR pathway, the nuclear import of the R2TP components Pih1p and Tah1p by Crm1p and Kap121p in *Saccharomyces cerevisiae* [150]. As the R2TP is necessary for Nop58p stability, this mechanism coordinates cell growth with C/D snoRNP assembly. Other proteins could modulate biogenesis. This is the case of the human H3K27 methyltransferase EZH2 that reinforces the FBL-NOP56 interaction by directly interacting with both proteins [151,152], whereas the RNA-binding protein NPM1 is suspected to modulate biogenesis with pathological consequences in dyskeratosis congenita [153]. However, for the latter, the mechanistic evidence are not clear yet. EZH2 illustrates the emerging link between C/D snoRNP assembly and chromatin effectors nicely. In *Saccharomyces cerevisiae*, the assembly factor Bcd1p interacts directly with the histone chaperone Rtt106p to modulate its recruitment at active genes [154]. Rsa1p with its partner Hit1p contribute to rDNA compaction by modulating Condensin accessibility [155]. In addition, the R2TP subunits RUVBL1 and RUVBL2 are components of several chromatin modification complexes, including the NuA4 histone acetyltransferase complex [156] and the chromatin remodeling complexes SWR1 [157] and INO80 [158]. C/D snoRNP assembly factors are playing other cellular functions, e.g., the R2TP provides a platform for the assembly and maturation of multiple protein complexes such as U4 snRNP-specific proteins, PIKK or RNA polymerase complexes (for a review, see [159]), and the assembly factor Rsa1p is required for the loading of Rpl10p onto the 60S subunit [160]. While these interactions between snoRNP biogenesis and different regulatory systems theoretically permit coordination, this possibility remains largely to be tested.

## 7. The Strange Case of Alternative C/D snoRNPs

An intriguing question regarding the regulation of C/D snoRNP biogenesis concerns the possibility of generating RNPs with different protein compositions. Some studies using fractionation and pull-down assays have proposed the presence of snoRNAs in complexes with abundant nuclear proteins such as splicing factors but devoid of FBL [161,162]. However, the particles have not been clearly identified yet, and several caveats remain to be evacuated. Co-fractionation does not mean association, and the absence of FBL detection does not mean absence of the protein. In addition, whether the snoRNAs identified correspond to precursors or degradation fragments has not been systematically analyzed. The existence of alternative RNPs clearly deserves supplementary characterization, but if confirmed, it would be particularly interesting to identify whether these complexes are diverted from conventional snoRNP biogenesis or degradation paths. For what is known currently, the first RNA determinant of snoRNP biogenesis is the K-turn formed by the C and D boxes that are recognized by SNU13. Then, the possibility for a snoRNA to assemble with an alternative set of proteins suggests either that (i) after recognition by SNU13 and at any step of the biogenesis, the assembly process is diverted towards a different set of proteins that ultimately competes with conventional core proteins or (ii) an RNA-binding protein other than SNU13 interacts specifically with the K-turn or another snoRNA feature, allowing the biogenesis of the alternative snoRNP. Alternatively, a remodeling process leading to protein exchange may intervene on a mature C/D snoRNP, e.g., in substitution of its recycling. Thus, in addition to fine biochemical characterization, it would be important to test the dependence of these RNPs on conventional assembly factors and core proteins.

## 8. The Diversity of C/D snoRNA Expression

By virtue of their role in the highly coordinated process of ribosome production, snoRNAs have long been considered housekeeping actors of cell function. In agreement, their expression largely correlates with the expression of other players of ribosome synthesis and translation. In Ascomycetes, the transcription of box C/D snoRNA genes is controlled by a Homol-D box that also controls the transcription of ribosomal protein genes [163], thus providing coordination [164]. Several genes coding for snoRNP components or related regulators is under the control of the transcription factor MYC in *Drosophila* and Human [165], which also controls numerous genes involved in ribosome biogenesis and protein synthesis [166]. In Metazoans, most snoRNA genes are located in introns of coding genes that exhibit a bias towards nucleolar function, ribosome structure, and protein synthesis [167]. In complement, several lncRNA genes that host snoRNA genes belong to the 5′TOP (5′-terminal oligopyrimidine) gene family [168,169] characterized by high-level transcription and growth-dependent regulation [170]. In Human, transcription of the few independent snoRNA genes is linked to cell proliferation by the transcriptional cofactors and RNA helicases DDX5 and DDX17 that recruit the histone deacetylase HDAC1 at promoter regions [171,172]. Yet, a more complex expression pattern is emerging from the numerous transcriptomic analyses accumulated in recent years. This is especially prominent in cancer as biopsies typically show altered expression of subsets of snoRNA genes [173,174,175] (for a review, see [176]) and have suggested their potential as diagnostic or prognostic markers [176]. Hence, several snoRNAs have been proposed as proto-oncogenes or tumor suppressors based on in vitro analyses [177,178,179,180,181]. They are also actors of other conditions, e.g., the Prader Willi syndrome (PWS) for the snord116 snoRNAs (for a review, see [182]), or the leukoencephalopathy with brain calcifications and cysts (LCC) for the U8 snoRNA [183]. In addition, C/D snoRNA genes have been found in some viral genomes where they are expected to play specific functions as suggested by the v-snoRNA1 produced by the Epstein-Barr virus, whose expression is drastically enhanced during the lytic cycle [130]. If most viruses do not encode their own snoRNAs, C/D snoRNAs are recurrently deregulated upon viral infection, e.g., by the Chikungunya virus (CHIKV) [184], by the Murine Cytomegalovirus (MCMV) [185], or the porcine reproductive and respiratory syndrome virus (PRRSV) [186]. Moreover, gene-trap insertional mutagenesis testing 12 distinct viruses has also revealed that, among other host genes, several human snoRNA genes participate in viral replication [187] (for an extensive review of the functional interactions between viruses and C/D snoRNAs, see [188]). Apart from pathological conditions, it appears that the expression of snoRNAs could be uncoupled from the level of ribosome synthesis. In Nematode, some snoRNAs display developmentally variable expression [189], while several snoRNAs from *Arabidopsis* and Human have been reported to be circadian clock-regulated [190,191]. C/D snoRNAs could also exhibit tissue-specificity, e.g., a strong tropism towards cerebral expression for several mammalian-specific snoRNAs [192,193,194]. The emerging versatility in the expression of many C/D snoRNAs questions their role. Going back to the ribosome, a reflection of this variability may be seen in the variable methylation levels observed at certain rRNA positions in different tissues or cellular conditions, giving rise to the concept of the specialized ribosome [195]. Indeed, some recent reports point to variations in rRNA methylation levels during mouse development [196], during human tumorigenesis [197,198], or in cell line models [199]. It suggests that the expression and/or catalytic activity of C/D snoRNPs could be finely regulated for adaptations that are beginning to be described. In this line, modifications due to high levels of FBL in cancers have been linked to impaired translation fidelity [198], fueling the notion of ribosomopathy associated with elevated cancer risk (for a review, see [200]). Interestingly, a similar scheme likely applies to snRNA methylation levels that modulate mRNA splicing [201].

## 9. The Diversity of C/D snoRNA Molecular Partners and Targets

Accumulating data support the notion that C/D snoRNAs perform molecular functions beyond the conventional ones. First, snoRNAs can methylate a broader set of targets than expected. It includes human tRNAs, affecting their nucleolytic cleavage [202], while in Archaea, the methylation of tRNAs by sRNAs was already described two decades ago [203,204,205]. In addition, human C/D snoRNAs could direct mRNA methylation and hence modulate the translation level of their target [206]. Interestingly, these data suggest that eukaryotic C/D snoRNPs could perform functions outside their main sites of accumulation, i.e., the nucleolus and CBs, which was already suggested by the fact that scaRNAs exhibit snRNA modification activity in the nucleoplasm in the absence of CBs [207]. Conversely, C/D snoRNPs target rRNAs in the nucleolus as expected but guide non-classical enzymatic activities, such as the yeast orphan snR4 and snR45 that promote rRNA acetylation catalyzed by Kre33p [208]. In addition, whether orphan or not, many snoRNAs have the potential to hybridize with cellular RNAs, including mRNAs as suggested by the in silico identification of energetically stable complementarities [209,210]. In agreement, studies using human cell lines have confirmed that the downregulation of specific snoRNAs affects in various ways mRNA targets, e.g., the modulation of steady-state levels by snord83b [211], of splicing by snord115 [212] and snord27 [162], or editing modulation by snord115 [213,214]. However, there is still no clear demonstration of the physical interaction between endogenous snoRNAs and their mRNA targets. It has to be mentioned that this could be a difficult task as the interaction may be limited in space and time, e.g., if occurring co-transcriptionally during pre-mRNA synthesis. Mechanistically, it could be supposed that C/D snoRNP binding induces steric hindrance and/or perturbates co-transcriptional pre-mRNA maturation processes. However, other mechanisms closer to conventional functions, such as RNP chaperoning, could also be at work, which deserves to be clarified.

A growing list of proteins has been identified to associate with C/D snoRNPs and to modulate their activity. Several of these interactions concern nucleolar proteins. In the nucleolus, C/D snoRNPs interaction with rRNA is controlled by factors modulating the affinity and specificity of the interaction. The RNA helicases HAS1 [215], DHR1 [216], and DHX37 [217] trigger the release of the U14 and U3 snoRNPs, respectively, from the ribosomal particle. The yeast ATPases Dbp3p or Prp43p promote rRNA methylation likely by favoring the recycling efficiency of C/D snoRNPs [218,219]. Concerning scaRNAs, the RNA-binding protein LARP7 increases the efficiency of methylation-guided activity by bridging scaRNAs with U6 snRNA, and its deficiency is associated with splicing alterations in Alazami syndrome [220]. Very interestingly, many other interactions with functional outcomes have been identified in the last years. The SNORD50A-SNORD50B locus produces snoRNAs that interact with the FIP1 subunit of the cleavage and polyadenylation specificity factor (CPSF) to modulate mRNA 3′-end processing [221], with the proto-oncogene KRAS to inhibit its activity [222,223] or with the E3 ubiquitin ligase TRIM21 and its substrate Guanosine 5′-monophosphate synthase (GMPS) to increase their interaction [224]. Numerous C/D and H/ACA snoRNAs have been proposed to interact with the poly-ADP-ribosyltransferase PARP1 independently of DNA damage and to stimulate its catalytic activity in the nucleolus, leading to ADP-ribosylation of the RNA helicase DDX21 and increased rDNA transcription [225]. The RNA-binding protein FMRP has been shown to interact with a subset of C/D snoRNPs, and its downregulation affects rRNA methylation [226], which might also be due to its interaction with ribosomal proteins [227,228]. The interaction of the RNA-binding protein TDP43 with a subset of C/D scaRNAs is necessary for their localization to CBs instead of the nucleolus, and its downregulation induces defects in methylation of U1 and U2 snRNAs and alters splicing [112].

It is very likely that other molecular partners are waiting to be identified. Indeed, several functional analyses point to C/D snoRNAs, whereas the underlying mechanisms are not identified yet. As examples, cellular cholesterol homeostasis is affected by snord60 expression, while the analysis of rRNA modifications did not identify changes in methylation levels [229]. The expression and cytoplasmic localization of three snoRNAs hosted by the ribosomal Rpl13a gene is increased on cells exposed to fatty acids, and their knockdown protects against lipotoxic and oxidative stress [230]. If the mechanism used by these snoRNAs is unknown, it has been shown that their nuclear export involves the nuclear export factor NXF3 [231]. More largely, the export to the cytoplasm of snoRNAs might be a recurrent response to cell stress as it has also been observed in the Silkworm [232]. Even more surprisingly, it has been proposed that snoRNAs could be secreted through exosomes and absorbed by distant tissues where they could perform 2′-O-methylation of rRNA [233]. It is not known how this complex journey is achieved and regulated.

Despite this burst of data, numerous C/D snoRNAs have no identified role even if molecular associations have been identified. This is the case for several C/D snoRNAs that interact with splicing factors [234,235] or the spliceosome [236] in human cells or with the chromatin factor DF31 that localizes to euchromatic regions in *Drosophila* and tethers a heterogeneous pool of short RNAs that is enriched in snoRNAs [237]. This is also true for RNA partners, as the complex repertoire of snoRNA-C/D RNA interactions is beginning to be revealed by large-scale approaches [238]. In addition, future works are required to identify the functions of most orphan snoRNAs. This category concerns around 30–50% of C/D snoRNAs in Mammals, whereas orphan H/ACA snoRNAs are much rarer (for a review, see [239]). The leading example in this category is the repeated snord116 genes whose loss of expression is suspected of promoting the Prader Willi syndrome [240,241,242,243,244,245,246,247]. To note, the existence of orphan snoRNAs is not restricted to Mammals, e.g., if only a few snoRNAs are concerned in *Saccharomyces cerevisiae*, a quarter of snRNAs in the Archaea *Pyrobaculum* are orphans [248]. In a minority of cases, a molecular function has been found, such as eutherian snord115 that controls splicing and editing of a target mRNA [212,213,214] or the yeast snR4 and snR45 that guide rRNA acetylation [208]. The latter are also interesting as it shows that the role of orphan snoRNAs could rely to conventional target.

## 10. Conclusions

As it is true for other ncRNAs, the snoRNA category is currently subjected to numerous and exciting developments despite its early discovery. This is especially true for C/D snoRNAs that, in addition to be leading actors of rRNA and snRNA maturation, appear now as regulators of other cellular RNAs, including mRNAs. In parallel, it emerges that the biogenesis and function of C/D snoRNPs are controlled by regulatory systems that are poorly described. In the face of the bloom of C/D snoRNA fates and functions, the field is in need of molecular dissection as currently, too few mechanisms are in hands to understand this overt variety. Importantly enough, it has to be stressed that the great majority, not to say all, snoRNAs accumulate dramatically in the nucleolus at a steady-state (or in the CBs for scaRNAs), e.g., including orphan ones. Consequently, a rigorous analysis of C/D snoRNA functions should always question the existence of direct or indirect effects in these compartments. As a corollary, it is arguable that new snoRNA roles remain to be discovered in these compartments. Altogether, these important efforts are necessary to build a renewed understanding of C/D snoRNA functions.
